# Preclinical Testing of Erlotinib in a Transgenic Alveolar Rhabdomyosarcoma Mouse Model

**DOI:** 10.1155/2011/130484

**Published:** 2011-04-20

**Authors:** Jinu Abraham, Laura D. Nelon, Courtney B. Kubicek, Aoife Kilcoyne, Sheila T. Hampton, Lee Ann Zarzabal, Francis J. Giles, Joel E. Michalek, Brian P. Rubin, Charles Keller

**Affiliations:** ^1^Pediatric Preclinical Testing Initiative, Pediatric Cancer Biology Program, Department of Pediatrics, Oregon Health & Science University, 3181 S.W. Sam Jackson Park Road, Portland, OR 97239-3098, USA; ^2^Greehey Children's Cancer Research Institute, The University of Texas Health Science Center, San Antonio, TX 78229, USA; ^3^Department of Medicine, The University of Texas Health Science Center, San Antonio, TX 78229, USA; ^4^Department of Epidemiology and Biostatistics, The University of Texas Health Science Center, San Antonio, TX 78229, USA; ^5^Department of Anatomic Pathology, Taussig Cancer Center and the Lerner Research Institute, Cleveland Clinic, Cleveland, OH 44195, USA

## Abstract

Rhabdomyosarcoma is an aggressive childhood malignancy, accounting for more than 50% of all soft-tissue sarcomas in children. Even with extensive therapy, the survival rate among alveolar rhabdomyosarcoma patients with advanced disease is only 20%. The receptor tyrosine kinase Epidermal Growth Factor Receptor (EGFR) has been found to be expressed and activated in human rhabdomyosarcomas. In this study we have used a genetically engineered mouse model for alveolar rhabdomyosarcoma (ARMS) which faithfully recapitulates the human disease by activating the pathognomic Pax3:Fkhr fusion gene and inactivating p53 in the maturing myoblasts. We have demonstrated that tumors from our mouse model of alveolar rhabdomyosarcoma express EGFR at both the mRNA and protein levels. We then tested the EGFR inhibitor, Erlotinib, for its efficacy in this mouse model of alveolar rhabdomyosarcoma. Surprisingly, Erlotinib had no effect on tumor progression, yet mice treated with Erlotinib showed 10–20% loss of body weight. These results suggest that EGFR might not be an *a priori* monotherapy target in alveolar rhabdomyosarcoma.

## 1. Introduction

Alveolar rhabdomyosarcoma (ARMS) is an aggressive soft-tissue sarcoma with myogenic features that has a very poor prognosis in children because of high metastatic potential and poor response to chemotherapy [1]. The survival rate for children with metastatic alveolar rhabdomyosarcoma is dismal even with the tremendous improvements in multimodality treatment [2, 3]. Recent studies have shown that molecularly targeted therapies can be very successful in treating malignant diseases like chronic myelogenous leukemia (CML) and gastrointestinal stromal tumors [4, 5]. Epidermal growth factor receptor (EGFR) is a receptor tyrosine kinase that has been shown to be expressed or activated in 32–50% of alveolar and 31–55% of embryonal rhabdomyosarcoma [6, 7]. Furthermore, the tyrosine-kinase inhibitor Erlotinib inhibits EGFR and is an FDA approved drug for the treatment of advanced non-small cell lung carcinoma [8, 9]. In the current study we have tested the preclinical efficacy of Erlotinib in treating alveolar rhabdomyosarcoma (ARMS) using a genetically engineered mouse model of ARMS.

## 2. Methods

### 2.1. Mice, Drug Administration, and Tumor Monitoring

All animal procedures were conducted in accordance with the Guidelines for the Care and Use of Laboratory Animals and were approved by the Institutional Animal Care and Use Committee (IACUC) at the University of Texas Health Science Center at San Antonio and the Oregon Health & Science University. The *Myf6Cre*,* Pax3:Fkhr*,* p53* conditional mouse model of alveolar rhabdomyosarcoma has been described previously [10–12]. Erlotinib was purchased from LC Laboratories (Woburn, MA, USA). Erlotinib was dissolved in sodium carboxymethylcellulose (0.3% weight/volume) and Tween 80 (0.1% volume/volume) in saline and administered at a dose of 100 mg/kg once daily by oral gavage for up to 2-3 weeks unless tumor size or the condition of the mice precluded ongoing treatment.

### 2.2. Mouse Primary Tumor Cell Cultures

 Fresh tumor tissues from mice were cut into small pieces and suspended in Dulbecco's modified Eagle's medium (DMEM) containing collagenase (1 mg/mL) at 37°C for 12 hours. The collagenase-containing medium was then removed, and the dissociated tumor cells were plated in fresh DMEM supplemented with 10% fetal bovine serum, penicillin (100 U/mL), and streptomycin (100 *μ*g/mL; Invitrogen) at 37°C with 5% CO_2_ in the incubator.

### 2.3. RT-PCR

RNA was extracted from tumor samples using TRIzol according to the manufacturer's instructions (Invitrogen, Carlsbad, CA, USA). The Qiagen RNeasy miniprep cleanup kit was used to purify the extracted RNA as per manufacturer instructions (Qiagen, Valencia, CA, USA). Reverse transcription using oligodT primers to generate cDNA was performed as previously described [11]. Real-time PCR was then performed on an ABI Prism 7500 Fast Real-Time PCR system using SYBR Green PCR Master mix according to the manufacturer's recommendations (Applied Biosystems, Foster City, CA, USA). Primer sequences for *EGFR* were as follows: 5′-CAGATGGATGTCAACCCTGAAG-3′ and 5′-TGGAGAGTGTGTCTTTAAATTCACC-3′.

### 2.4. Western Blotting

Total protein was extracted from naïve and Erlotinib-treated primary tumor cell cultures by homogenization using radioimmunoprecipitation (RIPA) buffer supplemented with phosphatase and protease inhibitors (Thermo Fisher Scientific, Waltham, MA, USA). Cell lysates were then centrifuged at 13,000 rpm for 10 minutes, and supernatant was used for western blot analysis using an anti-EGFR and anti-phospho-EGFR (Tyr845) antibody (Cat no. 2232 and no. 2231, Cell Signaling Technology, Beverly, MA, USA). Appropriate peroxidase-conjugated secondary antibodies (Vector Laboratories, Burlingame, CA, USA) were used at 1 : 5000 dilution. Chemiluminescence was then detected using SuperSignal West Pico Chemiluminescent Substrate or SuperSignal West Dura Extended Duration Substrate (Pierce Biotechnology, Rockford, IL, USA).

### 2.5. Histopathology

Histology and Myogenin/Desmin immunohistochemistry were performed to affirm diagnosis for each tumor, as previously described [12].

### 2.6. Statistical Analysis

The alveolar rhabdomyosarcoma (ARMS) and skeletal muscle (SKM) groups were contrasted with regard to the mean *Egfr* mRNA expression with a *t*-test. Erlotinib-treated (*n* = 13) and untreated control (*n* = 12) groups were contrasted with regard to the mean tumor volume (cc), measured repeatedly every 2 days for 19 days or until death, using a repeated measures linear model with an autoregressive (1) covariance structure with an adjustment for day, treatment (Erlotinib versus Control), initial tumor size (small: <0.4 cc, large: >0.4 cc), and the treatment by day interaction. The interaction of day by treatment was not significant and was removed from the model. Weight was measured repeatedly every 2 days for 15 days or until death, and treatment groups were contrasted on the mean weight with a repeated measures linear model with an auto regressive (1) covariance structure with an adjustment for day, treatment (Erlotinib versus Control), and the treatment by day interaction. The day by treatment interaction was not significant and was removed from the model. All statistical testing was two-sided with a significance level of 5% and was performed with SAS Version 9.2 for Windows (SAS Institute, Cary, North Carolina).

## 3. Results

### 3.1. Although EGFR Is Expressed in Murine aRMS, Erlotinib Has No Therapeutic Effect

**Figure 1:**
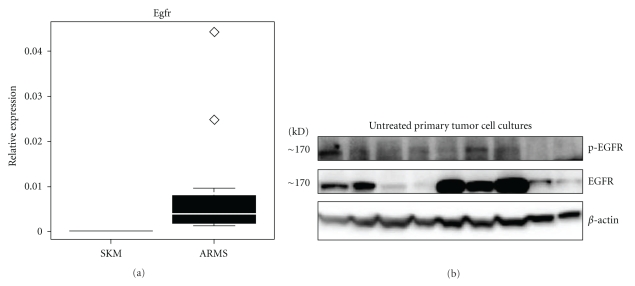
High expression of Egfr in mouse alveolar rhabdomyosarcoma. (a) Quantitative RT-PCR represented as a box and whisker plot showing expression of *Egfr* in skeletal muscle and alveolar rhabdomyosarcoma from the mouse model. (b) Western blots showing the expression and phosphorylation of Egfr in primary cell cultures established from untreated tumors from the mouse model.

**Figure 2:**
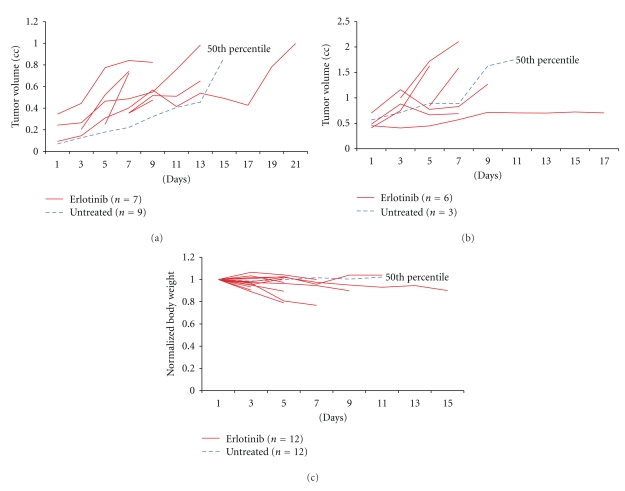
Tumor growth and weight loss in rhabdomyosarcoma-bearing mice treated with Erlotinib. (a) Tumor growth over time in mice which had tumors smaller than 0.4 cc at diagnosis. The 50th percentile represents *n* = 9 control (untreated) tumor-bearing mice. (b) Tumor growth over time in mice which had tumors larger than 0.4 cc at diagnosis. The 50th percentile represents *n* = 3 control (untreated) tumor-bearing mice. (c) Animal body weights over the duration of therapy.

**Figure 3:**
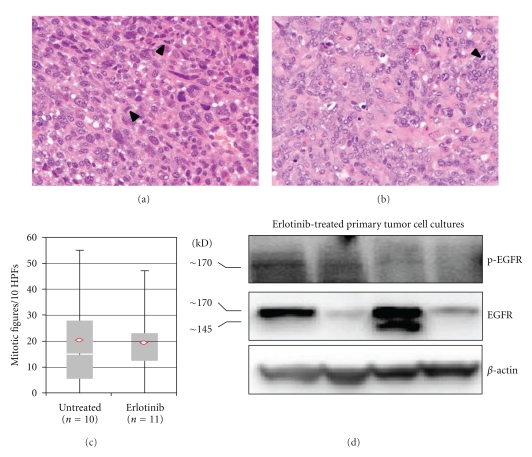
Histological and biochemical analysis of untreated and Erlotinib-treated tumors. Representative histology of (a) untreated and (b) Erlotinib-treated tumors. (c) Mitotic figures/10 high-power fields for untreated and Erlotinib-treated mice (*P* = 0.841). (d) Western blots showing the expression and phosphorylation of Egfr in primary tumor cell cultures from Erlotinib-treated mice.

To validate our mouse model of alveolar rhabdomyosarcoma for the study of EGFR inhibition, the expression of *Egfr* mRNA levels in skeletal muscle and murine tumors was compared using quantitative RT-PCR. The mean expression (mean ± SD) of *Egfr* mRNA was significantly higher in alveolar rhabdomyosarcoma compared to normal skeletal muscle (*P* < 0.001; Figure 1(a)). Western blot analysis showed stochastic expression of Egfr protein in 8 of 9 primary tumor cell culture lysates, and activation (phosphorylation) of EGFR was observed in 5 of 9 naïve (untreated) primary tumor cell cultures (Figure 1(b)). For *in vivo* EGFR inhibition studies, the structure and purity of Erlotinib were verified by mass spectrometry at the proteomics core facility of the University of Texas at San Antonio (data not shown). The highest tolerated dose of Erlotinib was taken from a prior preclinical study [13]. Tumor-bearing mice were thus treated with 100 mg/Kg Erlotinib daily by oral gavage. The 13 mice treated with Erlotinib did not respond to treatment. The Erlotinib and untreated control groups did not differ significantly with regard to mean tumor volume (*P* = 0.21; Figures 2(a) and 2(b)). Mice treated with Erlotinib experienced a 10–20% loss in body weight with no corresponding weight change in controls (*P* = 0.045; Figure 2(c)). Microsteatosis and vacuolar changes of the liver were also seen in two Erlotinib-treated mice (data not shown). No significant difference was found between the mitotic frequency for untreated and Erlotinib treated tumors (Figures 3(a), 3(b), and 3(c)). Primary cell cultures from Erlotinib-treated tumors, like untreated tumors, showed stochastic expression and activation (phosphorylation) of EGFR (Figure 3(d)).

## 4. Discussion

 Erlotinib (Tarceva) is a highly potent inhibitor of the tyrosine-kinase receptor Egfr [14]. Erlotinib has been shown to have single-agent antitumor activity in preclinical models of non-small cell lung carcinoma, yet when used in combination with chemotherapeutic agents like cisplatin or gemcitabine, the antitumor activity was significantly enhanced [9]. Similarly, in a phase III clinical trial involving patients with advanced pancreatic cancer, when Erlotinib was administered in combination with gemcitabine, a 23.5% increase in survival rate was observed compared to patients who received gemcitabine alone [15].

In our study, surprisingly, Erlotinib had no effect on *in vivo* tumor growth despite the presence of EGFR and the ability of tumors to phosphorylate this receptor tyrosine kinase. This result suggests that Egfr might not be an optimal therapeutic target for single-agent treatment of ARMS. A possible limitation of our study is that Erlotinib may not have been absorbed well orally, and hence the drug might not have reached the tumors at the required concentrations (pharmacodynamic studies were not performed here). On the other hand, at the dosage tested, dose-limited weight loss was observed. Toxicity at this dose range is consistent with a Children's Oncology Group Phase I study of Erlotinib with 85–110 mg/kg/day, for which the dose-limiting toxicities were rash, hyperbilirubinemia, and diarrhea [16]. Another reason for the lack of activity of Erlotinib may have been that Egfr might heterodimerize with Her2, raising the possibility that targeting the Egfr:Her2 heterodimers might be a better therapeutic strategy in alveolar rhabdomyosarcoma. Similarly, while Egfr homodimers may not play a tumor maintenance role in previously untreated alveolar rhabdomyosarcoma, the possibility remains that refractory (chemotherapy or kinase inhibitor resistant) tumors may still in fact be driven by Egfr tyrosine kinase receptor signaling. Taken together, we suggest that for future Phase II regimens Egfr may still be a worthwhile target of investigation in the context of combination therapy for refractory alveolar rhabdomyosarcoma- although, of limited value as monotherapy.

##  Conflict of Interests

The authors have no conflict of interests or financial disclosures.
